# Supplementary material for: Chameau (HBO1) regulates starvation resistance in Drosophila melanogaster in a temperature-dependent manner
Source: Life Sci Alliance. 2025 Nov 6;9(1):e202503524. doi: 10.26508/lsa.202503524 (PMC12592709; doi:10.26508/lsa.202503524)
Supplement: Supplementary file 21 [file LSA-2025-03524_TableS16.pdf]

Sample Name	1,3-bisphosphoglycerate/2,3-diphosphoglycerate	3-phosphog	Acetyl-CoA
chm-23-0hr	1.92	0.64	0.81
chm-23-0hr	0.00	0.53	0.92
chm-23-0hr	0.00	0.14	1.53
chm-23-0hr	0.00	0.21	1.59
chm-23-0hr	0.00	0.75	1.27
chm-23-0hr	0.00	2.54	0.50
chm-23-0hr	1.66	1.47	0.63
chm-23-0hr	1.34	0.00	1.14
chm-23-24hr	2.56	0.08	0.99
chm-23-24hr	0.00	1.41	1.27
chm-23-24hr	0.00	0.54	0.60
chm-23-24hr	0.00	0.95	0.27
chm-23-24hr	0.00	0.79	1.62
chm-23-24hr	0.00	2.00	0.23
chm-23-24hr	1.59	1.19	1.13
chm-23-24hr	0.00	2.37	0.48
chm-25-0hr	1.60	0.64	0.64
chm-25-0hr	1.19	0.85	0.47
chm-25-0hr	1.17	0.97	0.89
chm-25-0hr	4.37	0.60	0.47
chm-25-0hr	5.61	0.85	0.97
chm-25-0hr	2.48	0.15	0.32
chm-25-0hr	0.00	1.07	0.66
chm-25-0hr	5.17	0.42	0.64
chm-25-24hr	0.00	0.55	1.35
chm-25-24hr	1.32	0.61	0.85
chm-25-24hr	0.00	0.25	0.62
chm-25-24hr	0.00	0.79	1.88
chm-25-24hr	5.90	0.49	0.82
chm-25-24hr	2.20	0.82	1.13
chm-25-24hr	0.60	1.18	1.33
chm-25-24hr	0.00	2.39	0.46
wt-23-0hr	0.00	0.10	4.11
wt-23-0hr	0.00	0.72	1.37
wt-23-0hr	1.75	0.97	0.58
wt-23-0hr	2.35	0.31	5.81
wt-23-0hr	0.00	3.01	4.27
wt-23-0hr	0.00	1.63	0.46
wt-23-0hr	3.28	0.70	1.10
wt-23-0hr	0.00	0.51	0.53
wt-23-24hr	0.00	0.06	1.05
wt-23-24hr	0.00	0.91	0.80
wt-23-24hr	0.00	3.00	0.79
wt-23-24hr	2.36	0.00	0.90
wt-23-24hr	0.00	1.19	0.39
wt-23-24hr	0.00	2.46	1.31
wt-23-24hr	0.00	1.01	1.47
wt-23-24hr	0.00	1.92	0.80

wt-25-0hr	0.00	3.97	1.25
wt-25-0hr	0.00	3.18	3.07
wt-25-0hr	0.00	0.36	0.96
wt-25-0hr	0.00	0.48	2.31
wt-25-0hr	3.80	1.41	4.41
wt-25-0hr	0.00	1.49	1.89
wt-25-0hr	0.00	3.55	0.54
wt-25-0hr	1.78	0.00	0.67
wt-25-24hr	0.00	0.04	0.21
wt-25-24hr	0.00	0.00	0.47
wt-25-24hr	7.78	4.84	0.82
wt-25-24hr	0.00	2.54	0.89
wt-25-24hr	3.99	0.16	0.37
wt-25-24hr	4.50	1.40	1.14
wt-25-24hr	2.50	0.13	0.37
wt-25-24hr	2.18	0.09	0.56

adenosine-!	adenosine-!	adenosine-!	Alpha-Keto	Aspartate	beta-hydro:	Citrate	CoASH	fructose-6-|
0.35	0.60	0.00	0.36	0.32	0.00	0.45	0.30	0.71
1.04	1.39	0.00	1.13	1.21	0.82	0.50	1.36	1.42
0.73	1.16	0.00	5.01	0.58	0.00	1.21	0.58	1.58
0.62	1.15	0.00	0.80	1.48	1.06	0.29	1.12	1.02
0.62	0.22	0.00	4.27	1.04	1.39	0.65	1.95	1.08
0.26	0.11	0.00	0.27	0.90	1.68	0.72	0.89	2.24
0.58	1.91	0.00	0.68	1.01	0.75	0.76	1.14	1.53
0.61	0.88	0.00	2.56	1.23	0.88	0.56	0.82	0.86
1.27	1.34	0.00	1.27	1.72	0.54	0.54	1.47	0.80
0.85	0.40	0.00	4.77	0.51	1.48	0.23	0.77	1.10
1.73	1.00	0.00	0.16	0.47	0.58	0.18	0.79	1.79
1.58	0.30	0.00	0.17	1.55	1.74	0.74	0.91	0.95
0.56	0.47	0.00	0.26	0.71	1.07	0.46	1.16	0.51
0.81	0.66	0.00	0.13	0.74	1.51	0.88	0.97	1.16
0.96	1.22	0.00	0.27	0.94	0.35	0.92	1.87	0.86
1.07	1.48	0.00	0.27	0.66	1.14	0.83	0.36	1.05
0.99	1.23	0.00	0.80	1.08	1.39	0.69	0.87	1.55
0.59	0.83	0.00	0.30	0.53	0.58	0.13	1.15	0.93
0.69	1.46	0.00	0.38	0.84	0.92	0.80	1.29	1.05
0.84	0.77	0.00	0.44	0.46	0.58	0.40	0.53	1.20
0.62	0.65	0.00	0.33	0.67	0.75	1.04	0.59	0.87
0.46	0.05	0.00	0.40	0.91	1.08	0.37	1.40	0.63
0.77	1.16	0.00	1.47	0.72	0.92	0.69	0.72	1.51
1.10	0.72	0.00	0.36	0.82	1.26	0.66	0.60	1.76
1.59	1.00	0.00	1.15	0.92	1.53	1.38	0.81	0.51
1.24	0.77	0.00	0.55	1.41	0.86	1.53	1.29	0.78
0.66	0.50	0.00	1.21	0.58	1.13	0.58	1.14	0.54
0.87	0.06	0.00	4.08	0.82	1.88	1.65	1.20	0.72
0.96	0.42	0.00	0.52	0.83	1.66	2.07	1.50	0.45
1.10	1.38	0.00	0.40	1.06	0.59	1.80	0.78	0.64
1.36	1.37	0.00	1.92	0.61	0.78	1.32	0.84	0.57
1.31	1.04	0.00	0.27	0.71	1.61	0.93	0.68	0.92
0.71	0.77	0.00	2.69	1.07	1.16	0.79	0.88	1.29
0.75	1.77	0.00	1.09	1.67	0.63	0.36	0.76	1.47
1.31	2.26	0.00	1.91	0.57	0.44	0.40	0.51	2.59
1.01	1.82	0.00	0.63	0.64	0.87	0.62	1.06	2.00
0.95	1.04	0.00	4.55	1.12	0.65	0.34	1.27	1.33
0.31	0.40	0.00	0.20	0.34	0.83	0.23	0.36	1.22
0.94	1.51	0.00	1.16	0.65	1.37	0.17	0.95	2.15
0.59	0.56	0.00	0.14	0.52	0.95	0.27	1.05	0.95
1.19	1.37	0.00	0.25	0.95	0.73	1.32	1.35	0.69
1.00	1.84	0.00	0.83	1.28	0.64	0.72	1.76	0.99
0.74	0.74	0.00	0.85	0.80	1.15	0.57	0.90	0.83
0.99	0.26	0.02	1.17	1.71	1.61	0.34	0.81	1.43
1.19	1.60	0.00	2.24	0.54	1.05	0.34	0.69	1.39
1.32	1.43	0.00	0.17	0.59	1.29	0.93	0.46	1.18
0.56	0.60	0.00	0.17	0.61	0.40	0.91	0.91	0.81
1.07	2.19	28.65	0.35	0.68	1.01	0.68	0.50	1.31

0.95	2.37	21.95	0.25	0.95	1.48	0.65	0.72	2.46
1.22	2.67	0.00	0.12	1.26	0.86	0.23	0.84	1.91
0.60	0.78	0.00	0.81	0.69	0.65	0.37	1.63	0.88
0.68	0.56	0.00	0.49	0.61	0.99	0.28	1.09	0.76
1.04	1.88	0.00	0.58	0.89	0.53	0.84	0.48	2.11
0.49	0.12	0.00	2.71	0.71	1.59	0.77	1.05	1.04
1.04	1.64	0.00	0.78	0.53	0.82	1.18	0.51	1.82
0.59	0.35	0.00	0.18	0.90	0.97	0.29	0.70	0.63
0.53	1.00	0.00	1.13	1.09	0.60	0.35	1.19	0.54
0.77	0.00	0.00	0.93	0.88	2.27	0.60	0.81	0.37
1.71	2.78	0.00	0.56	0.87	0.98	1.39	0.82	1.52
0.72	0.38	0.00	4.31	0.62	1.43	0.98	1.27	0.73
0.63	0.00	0.00	0.56	2.04	1.36	0.64	1.56	0.71
1.31	1.47	0.00	0.98	0.65	1.25	0.52	1.04	1.20
0.87	2.05	0.00	2.28	1.15	1.56	0.52	0.86	1.04
0.76	1.32	0.00	0.44	1.09	0.56	0.62	1.50	0.56

Fumarate	Glutamate	Glutamine	Iso/Leucine	Lactate	malate	malonyl-Co	Methionine	NAD+
0.65	0.44	0.31	0.46	0.37	0.46	0.00	0.48	0.38
0.17	0.63	1.00	0.76	0.66	0.46	1.71	0.89	0.83
0.58	1.04	0.71	0.97	0.59	1.30	0.00	0.85	0.77
0.00	0.67	1.36	1.27	0.78	0.33	0.63	2.13	1.01
0.25	0.75	1.72	1.79	1.25	1.16	0.51	3.49	0.69
1.08	1.08	0.47	2.71	0.87	1.15	0.49	1.52	1.02
0.23	0.80	1.46	1.25	0.82	0.61	0.00	0.67	0.77
0.23	0.80	0.97	1.65	0.80	0.41	0.62	0.00	0.75
0.65	0.63	1.49	0.61	0.78	0.58	0.55	0.47	0.92
0.00	0.91	0.41	0.63	1.45	0.67	0.34	0.70	1.11
0.53	0.54	1.03	0.33	2.03	0.86	1.25	0.00	1.23
0.97	1.39	1.43	0.59	1.73	0.00	0.60	0.00	1.27
1.58	0.42	1.16	0.89	1.05	0.75	0.19	5.03	0.70
0.00	0.94	0.97	0.65	1.39	0.84	0.37	1.59	0.77
0.38	0.75	2.33	0.81	2.07	0.54	0.51	1.78	0.56
1.38	1.26	0.68	0.59	1.47	1.12	0.84	1.56	1.48
1.00	1.06	1.00	1.45	1.47	1.26	0.81	0.79	1.33
0.13	0.66	0.64	1.68	0.46	0.22	2.96	2.59	0.71
0.70	0.81	1.33	2.92	0.76	0.77	1.99	5.65	0.86
1.33	0.60	0.40	2.33	1.38	0.82	0.91	2.57	0.91
2.45	1.31	1.00	1.58	0.72	0.86	0.00	1.05	1.05
0.00	0.98	0.55	2.72	0.51	0.40	0.80	0.66	0.49
1.20	0.97	0.82	1.71	0.99	1.49	0.78	1.63	1.02
1.07	1.18	1.13	1.02	2.11	1.05	0.53	1.10	1.49
0.51	1.22	1.41	0.90	0.74	0.90	0.33	0.43	1.21
0.96	0.94	0.91	0.71	2.15	1.48	0.21	0.79	1.07
1.22	0.97	0.75	0.73	1.01	0.90	1.08	0.00	0.74
1.46	1.75	0.41	0.91	0.91	2.11	4.31	0.00	0.86
0.00	1.31	0.65	0.93	0.78	1.60	0.88	0.00	0.81
0.83	0.71	0.77	0.61	0.69	1.15	1.03	1.23	0.98
0.65	1.10	0.65	0.46	0.80	1.04	1.51	0.00	1.08
0.53	1.02	0.69	0.59	1.24	1.02	2.82	0.00	1.34
0.53	1.13	0.89	2.36	1.03	1.25	1.74	0.82	0.60
0.29	0.62	2.28	0.84	0.65	0.79	0.59	1.02	0.74
0.75	0.97	1.08	1.44	0.87	0.83	0.95	0.00	1.61
1.04	0.80	1.54	1.26	1.49	0.68	1.31	1.66	1.57
0.00	0.61	0.58	1.72	1.38	0.39	1.68	1.35	1.30
0.26	0.48	0.46	1.11	0.48	0.60	1.74	0.73	0.61
0.00	1.63	0.80	1.47	0.83	0.86	1.10	1.42	1.63
0.71	0.77	0.32	1.96	1.78	0.56	2.35	3.32	0.77
1.54	0.94	1.66	0.44	1.33	1.42	0.00	0.89	0.72
0.68	0.76	1.64	0.58	1.12	0.61	0.64	0.61	0.85
1.45	1.28	0.55	0.75	0.99	0.81	1.44	0.00	1.16
0.75	0.59	1.23	0.46	1.84	0.66	0.34	0.00	0.78
0.66	0.91	0.88	0.53	0.55	0.59	0.65	0.00	1.39
1.18	1.01	1.31	0.67	1.31	1.11	0.66	1.10	1.12
0.00	1.21	1.13	0.88	1.31	0.82	0.42	1.58	1.13
1.59	1.01	1.14	0.58	1.40	0.76	0.00	0.54	1.27

0.73	1.49	1.27	1.32	0.98	0.98	1.95	1.93	1.55
0.33	0.98	1.27	2.37	2.46	0.67	1.13	2.52	1.28
0.23	0.67	0.96	3.33	0.94	0.31	0.28	4.83	0.48
0.63	0.93	0.87	2.24	1.35	0.50	0.00	2.02	0.65
0.83	1.13	1.48	1.68	1.55	1.02	0.51	2.79	1.07
1.44	1.04	0.47	3.62	1.09	1.39	3.40	1.06	0.78
1.20	0.66	0.85	1.01	0.72	0.76	0.77	1.43	1.00
0.42	0.50	0.86	1.48	1.24	0.37	0.31	1.44	0.41
0.00	0.61	0.92	0.46	0.37	0.35	1.11	0.26	0.67
0.76	1.13	0.77	0.59	0.82	1.38	0.00	0.00	0.64
0.94	1.16	1.61	0.86	0.74	1.15	0.00	0.93	1.78
1.60	1.14	0.37	0.88	1.08	1.40	1.84	0.00	0.79
0.20	0.59	0.74	0.46	0.53	1.04	2.09	0.00	0.53
1.31	1.19	0.84	0.61	1.24	0.74	0.29	0.66	1.83
0.35	0.76	1.07	0.49	0.74	0.47	0.48	2.00	1.07
0.24	0.98	0.73	0.98	0.65	0.52	0.40	1.86	0.84

NADH	NADP+	NADPH	Pyruvate	SAH	SAM	Sarcosine	Spermidine	succinate
0.00	0.51	0.44	0.00	0.00	0.51	0.75	0.00	0.37
0.00	0.75	1.25	0.06	0.00	0.00	1.65	0.00	0.74
3.53	1.09	0.27	0.01	0.00	1.02	0.45	0.00	0.92
0.00	0.82	1.36	0.11	0.00	1.05	1.87	0.00	1.04
0.00	0.90	1.56	0.09	0.00	0.00	1.16	0.00	1.22
0.00	2.14	0.64	0.02	0.00	0.00	1.43	0.00	0.94
0.00	0.77	1.58	0.09	0.00	0.75	1.53	0.00	0.91
0.15	0.60	1.04	0.04	0.00	0.38	1.40	0.00	0.78
0.00	0.94	1.49	0.07	0.00	0.00	1.61	0.00	0.98
0.00	1.30	0.51	5.64	0.00	1.35	1.08	0.47	0.79
4.12	1.00	0.56	0.05	0.00	0.81	0.21	0.00	0.94
0.00	1.23	2.21	0.01	7.00	1.76	0.19	0.00	0.50
0.00	0.87	0.50	5.23	0.00	1.50	0.94	0.00	0.71
0.55	1.04	0.42	4.80	0.00	0.00	1.22	0.00	1.05
0.00	0.76	1.27	2.90	0.00	0.00	1.38	0.00	1.07
0.00	1.50	2.07	4.37	0.00	0.00	0.89	0.00	0.88
0.00	1.15	0.75	0.16	0.00	1.53	0.39	0.00	1.08
0.13	0.59	1.01	0.06	0.00	0.00	1.28	0.25	0.57
0.00	0.74	1.40	0.11	0.00	0.00	1.90	0.00	0.96
0.00	1.26	0.79	4.33	0.00	0.00	0.82	0.00	0.72
0.19	0.84	0.30	0.05	0.00	0.00	1.16	0.63	0.86
0.00	0.48	0.80	0.02	0.00	1.07	0.79	0.00	0.51
0.00	1.13	1.17	0.03	0.00	1.35	1.50	0.00	0.78
0.00	1.27	0.78	0.03	0.00	0.00	0.26	0.69	1.04
0.23	1.32	0.43	0.10	0.00	0.00	0.84	0.00	1.07
0.77	0.86	0.25	1.18	0.00	0.00	1.81	0.00	1.13
0.00	0.78	0.00	3.30	0.00	0.00	1.00	0.00	0.87
1.77	1.12	1.17	0.02	0.00	0.00	1.25	0.00	1.08
2.15	0.84	1.04	0.02	0.00	0.00	0.31	0.00	1.11
0.00	0.74	1.02	0.24	0.00	1.53	1.78	0.00	1.04
0.00	1.12	0.27	0.15	0.00	0.00	1.40	0.49	0.86
2.53	1.13	0.20	0.12	0.00	0.00	0.84	0.00	0.96
0.00	0.86	0.76	0.03	0.00	0.00	2.39	0.00	0.95
0.58	0.68	0.96	0.06	0.00	0.00	1.13	0.00	0.88
0.00	1.70	0.99	0.01	0.00	0.00	0.73	25.92	1.01
0.00	1.40	1.01	0.04	0.00	0.00	1.82	0.00	1.63
0.00	1.39	2.39	0.14	0.00	1.62	1.10	0.00	0.98
0.00	1.02	1.60	0.00	26.32	0.00	0.54	6.99	0.70
8.76	1.77	0.00	0.01	0.00	0.00	0.77	0.00	0.93
0.00	0.79	0.00	6.26	0.00	0.86	1.84	0.00	0.75
0.00	0.71	1.17	0.04	0.00	0.00	0.93	0.00	1.08
0.00	0.89	1.34	0.09	0.00	0.00	0.65	0.00	1.27
0.00	1.62	1.91	1.32	0.00	1.65	1.74	0.00	0.85
0.00	1.10	2.05	0.93	0.00	0.00	0.27	0.00	1.17
0.17	1.35	0.38	0.02	6.37	0.00	0.70	0.00	0.85
4.88	1.44	1.75	4.51	0.00	2.20	1.02	3.67	1.08
4.44	1.04	0.25	5.97	0.00	0.00	0.94	0.00	0.75
5.92	1.45	3.02	2.99	0.00	4.82	0.96	42.74	0.83

6.29	1.70	3.47	0.01	0.00	2.14	1.11	0.60	1.16
0.00	1.64	1.80	5.82	0.00	4.43	1.38	0.00	1.29
0.00	0.53	1.07	4.64	0.00	0.00	1.67	0.00	0.95
0.00	0.65	0.25	5.67	0.00	0.91	2.17	0.00	0.78
4.49	1.39	2.90	0.10	6.61	0.00	1.34	1.15	1.26
0.00	1.69	2.92	0.00	0.00	0.00	0.31	0.00	0.96
3.72	1.01	0.75	0.05	0.00	1.45	0.71	0.00	0.72
0.00	0.67	1.36	2.29	0.00	1.54	0.22	0.00	0.70
0.50	0.51	0.93	0.09	0.00	0.00	1.19	0.00	0.80
0.00	0.48	0.55	0.08	0.00	3.32	0.00	0.00	0.96
5.99	1.68	3.13	0.08	0.00	4.18	0.54	0.00	1.39
0.00	1.29	0.14	0.01	0.00	0.00	0.72	0.00	1.02
0.27	0.47	0.61	0.04	0.00	1.34	0.56	0.00	0.64
0.00	1.27	0.32	0.10	0.00	0.00	1.68	0.00	1.09
0.00	0.95	1.55	0.61	0.00	4.00	1.85	0.00	0.98
0.00	0.63	1.18	0.11	0.00	0.78	0.59	0.00	0.74

Succinyl-Cc	Valine
0.24	1.02
0.38	1.90
0.84	0.00
0.36	6.96
1.88	0.00
2.28	0.00
0.24	4.96
0.99	4.28
1.02	2.43
0.45	0.00
0.84	0.00
0.19	0.00
0.74	0.00
0.00	0.00
0.89	3.06
0.15	0.00
0.76	0.00
0.88	0.00
0.61	0.00
1.67	0.00
0.29	2.71
1.29	0.00
1.29	2.92
0.00	0.00
1.75	0.00
0.33	0.00
0.83	0.00
0.76	0.00
1.13	0.00
2.35	0.00
0.52	0.00
1.08	0.00
2.08	0.00
0.60	2.10
2.70	5.73
0.86	3.55
0.28	2.45
1.33	0.00
0.27	3.20
0.12	0.00
1.36	0.00
1.91	1.76
1.61	0.00
0.48	0.00
0.31	0.00
0.17	0.00
1.05	0.00
0.23	2.53

2.15	8.40
1.79	3.32
0.73	0.00
0.66	3.56
0.87	9.61
2.14	0.00
1.14	3.63
0.40	8.43
0.86	0.00
0.86	0.00
0.00	1.93
3.54	0.00
1.64	0.00
1.22	0.00
0.56	0.00
0.75	0.00
